# Effects of attenuation map accuracy on attenuation-corrected micro-SPECT images

**DOI:** 10.1186/2191-219X-3-7

**Published:** 2013-01-31

**Authors:** Chao Wu, Hugo A Gratama van Andel, Peter Laverman, Otto C Boerman, Freek J Beekman

**Affiliations:** 1 Section Radiation, Detection & Medical Imaging, Delft University of Technology, Mekelweg 15, Delft, 2629 JB, the Netherlands; 2 Rudolf Magnus Institute of Neuroscience, University Medical Center Utrecht, STR 5.101, Univeriteitsweg 100, Utrecht, 3584 CG, the Netherlands; 3 MILabs B.V., Heidelberglaan 100, Utrecht, 3584 CX, the Netherlands; 4 Department of Nuclear Medicine, Radboud University Nijmegen Medical Centre, Geert Grooteplein 8, Nijmegen, 6525 GA, the Netherlands

**Keywords:** Molecular imaging, SPECT, CT, Small animal, Misregistration, Attenuation correction, Quantification

## Abstract

**Background:**

In single-photon emission computed tomography (SPECT), attenuation of photon flux in tissue affects quantitative accuracy of reconstructed images. Attenuation maps derived from X-ray computed tomography (CT) can be employed for attenuation correction. The attenuation coefficients as well as registration accuracy between SPECT and CT can be influenced by several factors. Here we investigate how such inaccuracies influence micro-SPECT quantification.

**Methods:**

Effects of (1) misalignments between micro-SPECT and micro-CT through shifts and rotation, (2) globally altered attenuation coefficients and (3) combinations of these were evaluated. Tests were performed with a NEMA NU 4–2008 phantom and with rat cadavers containing sources with known activity.

**Results:**

Changes in measured activities within volumes of interest in phantom images ranged from <1.5% (^125^I) and <0.6% (^201^Tl, ^99m^Tc and ^111^In) for 1-mm shifts to <4.5% (^125^I) and <1.7% (^201^Tl, ^99m^Tc and ^111^In) with large misregistration (3 mm). Changes induced by 15° rotation were smaller than those by 3-mm shifts. By significantly altering attenuation coefficients (±10%), activity changes of <5.2% for ^125^I and <2.7% for ^201^Tl, ^99m^Tc and ^111^In were induced. Similar trends were seen in rat studies.

**Conclusions:**

While getting sufficient accuracy of attenuation maps in clinical imaging is highly challenging, our results indicate that micro-SPECT quantification is quite robust to various imperfections of attenuation maps.

## Background

Small-animal single-photon emission computed tomography (micro-SPECT) plays an increasingly important role in biomedical research [1-9]. Attenuation correction, together with correction for effects such as scatter and camera blurring, is essential for obtaining highly quantitative SPECT images. Efforts have been made to improve quantitative accuracy in micro-SPECT. For instance, Hwang et al. [10] and Vanhove et al. [11] showed that by using attenuation maps derived from micro-CT data, cupping artefacts in micro-SPECT images could be eliminated and quantitative errors could be reduced with iterative attenuation correction [12] and window-based scatter correction [13] methods. We recently introduced an optical-contour-based modified Chang method [14] for attenuation correction in multi-pinhole SPECT, which is applied post-reconstruction and leads to small quantitative errors (1.7% and 2.1% on average in phantom and rat studies with ^99m^Tc, respectively) [15]. This method was later extended to a CT-based non-uniform attenuation correction and tested with four different isotopes on a micro-SPECT/CT system [16]. Average quantitative errors of 2.1%, 3.3%, 2.0% and 2.0% in activities of sources implanted in rat cadavers were achieved for ^125^I, ^201^Tl, ^99m^Tc and ^111^In, respectively.

In clinical SPECT, problems caused by inaccurate attenuation maps have been thoroughly investigated. For example, Tonge et al. [17,18] indicated that even minor degrees of misregistration (a couple of millimetres) between SPECT and CT can create significant artefacts. Goetze et al. [19] found that misregistration of less than 1 cm contributed significantly to changes in myocardial perfusion images. Studies of e.g. Kennedy et al. [20] found misregistration of more than 3.4 mm in 73% of clinical SPECT/CT studies. Such studies even raised the question whether attenuation correction should be applied to clinical myocardial perfusion SPECT. Results about attenuation map inaccuracies and their effects on quantitative accuracy for clinical SPECT cannot be directly translated to micro-SPECT studies because of e.g. higher resolution, different anatomies and less attenuation in micro-SPECT studies. Since anatomical images from external scanners are often combined with micro-SPECT, registration and scaling issues occur quite often. Therefore, knowledge about required attenuation map accuracy is highly important.

The aim of the present study is to investigate the effects of attenuation map imperfections on the accuracy of micro-SPECT quantification. Two main sources of attenuation map inaccuracies were emulated: (1) misalignments between micro-SPECT and micro-CT images, e.g. as could result from misregistration or animal movement; and (2) global errors in the attenuation coefficients derived from micro-CT data, e.g. as could result from sub-optimal CT calibration.

## Methods

Animal studies involved in this study were conducted following the regulations approved by the Animal Experiment Committee of the Radboud University Nijmegen.

### Phantom and animal imaging in U-SPECT-II/CT

**Figure 1:**
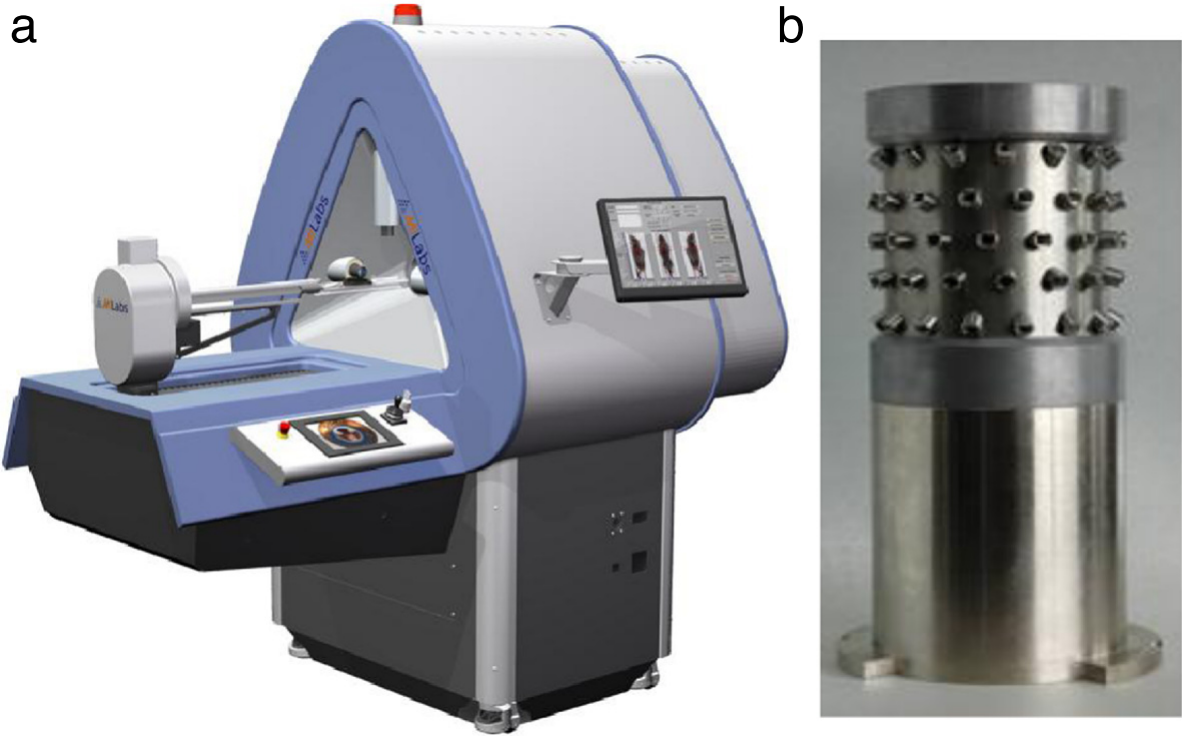
**Images of the system and rat collimator used in the present study.** (**a**) U-SPECT-II/CT multi-pinhole system and (**b**) rat collimator.

The U-SPECT-II/CT system used in the present study (MILabs, Utrecht, the Netherlands; Figure 1) comprises three stationary gamma cameras equipped with different cylindrical multi-pinhole collimators optimized for differently sized rodents, each containing 75 focused pinholes. An XYZ stage shifts the animal during acquisition for scanning large volumes up to the entire animal. The integrated high-throughput micro-CT comprises a fast circular cone-beam setup dedicated for low-dose scanning with an air-cooled metallo-ceramic tube (voltage range 20 to 65 kV) and a 1280 × 1024 pixel 12-bit CCD-based detector. Performance of this system is described in [15,16,21-25].

**Figure 2:**
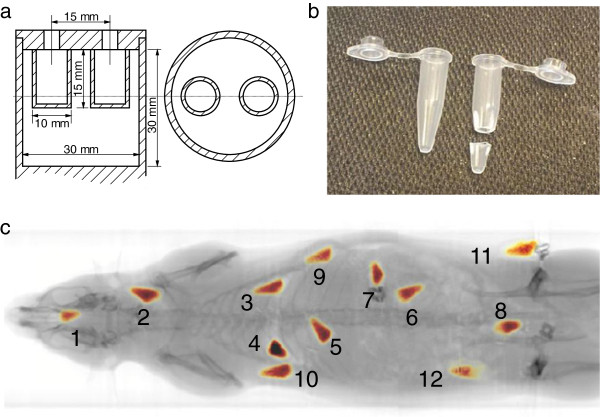
**NEMA image quality phantom, microcentrifuge tube and rat body showing**^**99m**^**Tc sources.** (**a**) Drawing of NEMA image quality phantom, (**b**) 0.5-ml microcentrifuge tube and its tip for making sources (sealed with Parafilm (Pechiney Plastic Packaging Company, Chicago, IL, USA) afterwards) used in the rat study and (**c**) rat with numbered ^99m^Tc sources with known activity.

A NEMA image quality phantom for small-animal imaging (NEMA NU 4–2008, NEMA, Rosslyn, VA, USA; Figure 2a) with two small air-filled chambers was first filled with ^99m^TcO_4_^−^ solution (8.65 MBq/ml). The activity was measured in a dose calibrator. Next, SPECT and fully circular CT (60 kV and 615 μA) were performed. Then, the same procedure was repeated for ^201^TlCl_3_, ^111^InCl_3_ and Na^125^I solutions, with activity concentrations of 5.90 MBq/ml, 5.91 MBq/ml and 4.54 MBq/ml, respectively. In addition, 12 small ^99m^Tc sources (approximately conical, about 6 mm in length and 3 mm in average diameter, and made from tips cut from 0.5-ml microcentrifuge tubes; Figure 2b) with known activity were inserted into a cadaver of a female Wistar rat surgically (inside or near the mouth, neck, left and right lungs, liver, stomach, left kidney, intestine, left and right shoulders, and left and right sides of the waist, as shown in Figure 2c). Total-body SPECT/CT was performed for the rat. This was repeated with three other rat cadavers for ^125^I, ^201^Tl and ^111^In. Activities of the ^125^I, ^201^Tl, ^99m^Tc and ^111^In sources ranged from 8.30 to 11.8 MBq, 3.82 to 7.23 MBq, 13.9 to 18.8 MBq and 6.02 to 11.7 MBq, respectively.

SPECT images were reconstructed by using the scanning focus method [26] combined with pixel-based ordered subset expectation maximization [27] using 16 subsets, 6 iterations and a 0.375-mm voxel grid, during which blurring and pinhole sensitivity effects were corrected [28] and an energy-window-based scatter correction method was implemented [13]. CT images were reconstructed with a 0.169-mm voxel grid, using NRecon (SkyScan, Kontich, Belgium) with its built-in modified Feldkamp algorithm and methods for beam hardening and ring artefact corrections. Both SPECT and CT images were rescaled with pre-measured calibration factors, and then SPECT images were registered to CT images by means of rigid transformations with pre-determined matrices.

More details on system calibration, phantom and animal preparation, data acquisition, image reconstruction, scatter correction and SPECT-CT registration have been described in [16].

### CT-based non-uniform attenuation correction for micro-SPECT

The Chang algorithm [14] is a practical attenuation correction method, which provides an approximation for obtaining the transmitted fraction (TF) for each voxel in reconstructed images. Being a post-reconstruction correction method, the Chang algorithm does not require generation of additional SPECT system matrices that each takes up tremendous amounts of memory and computation. Therefore, the Chang algorithm can be easily applied to images acquired from any micro-SPECT system. This algorithm has been shown to lead to highly accurate results [15,16] which can be attributed to the fact that (in contrast to clinical SPECT) only small corrections are required in rodents, justifying the use of this first-order method (in clinical SPECT, iterative attenuation correction is highly preferred while memory demands are different). Details about implementation of the modified Chang algorithm for multi-pinhole SPECT are found in [16]. In brief, the TF along every projection line from a certain voxel is calculated, and the average of all these TFs is treated as the overall TF for that voxel:

(1) $$\mathrm{TF}=\frac{1}{M}\sum_{m=1}^{M}\exp\left(-\int_{L_{m}}\mu \left(l\right)dl\right)\;\text{,}$$

where *M* is the number of projections involved in acquisition for data of a certain voxel, *L*_*m*_ denotes the *m*th projection path of gamma photons and *μ* is the attenuation coefficient on that projection line *L*_*m*_. Attenuation coefficients were derived from CT images by linear scaling [29]:

(2) $$\mu =\mu_{0}\left(\frac{\mathrm{HU}}{1,000}+1\right)$$

in which HU is the CT image intensity in Hounsfield units, and *μ*_0_ is the linear attenuation coefficient associated with water and the energy of the photons used in SPECT. *μ*_0_ was obtained using the NIST data tables [30]. In case that more than one photon energy was involved, *μ*_0_ was chosen to be the average of *μ*_0_(*E*) weighted by the number of photons detected under each energy *E*. When the TF for each voxel was estimated, the attenuation was compensated by dividing each voxel value by its corresponding TF [16].

### Attenuation correction with different attenuation maps

For each scan, an attenuation coefficient map was created from the registered CT image by linear scaling as in Equation 2 and then converted to a transmitted fraction map with the non-uniform Chang algorithm as described in Equation 1. To only evaluate the impact of inaccuracies in attenuation maps on the SPECT quantification, we considered these original attenuation maps to be accurate, and thus, we used the SPECT images corrected with these maps as reference images. Meanwhile, quantitative errors, i.e. differences between activities measured in images and in a dose calibrator, were also calculated for each reference image for reasons of comparison.

To investigate the effect of misregistration, we shifted the original TF maps for phantom studies in either + *x* (towards the right side of the system) or + *y* (downwards) direction and the maps for animal studies in + *x*, +*y* or + *z* (towards the back side of the system) direction. The distances of shifting in each direction were set to 1 mm and 3 mm. All original TF maps were also rotated anticlockwise by 15° to emulate the consequences of animal movement during scans. All these shifted and rotated TF maps were used for attenuation correction of their corresponding SPECT images. Later on, we globally changed the attenuation coefficients in the attenuation maps by ±10% of the original values. This was to emulate errors introduced by CT itself and/or from converting CT values to attenuation coefficients. TF maps were again calculated with the non-uniform Chang algorithm from these altered attenuation maps and then used for correcting for attenuation in their corresponding SPECT images. As a final test, the TF maps calculated with the +10% attenuation coefficient maps were also rotated anticlockwise by 15° and shifted by 3 mm in the *x* direction. This way, we introduced a combination of ‘worst-case’ errors. These TF maps were then employed for attenuation correction. All images that were corrected with the abovementioned inaccurate attenuation maps were compared to the reference images.

All the above operations were performed for ^125^I, ^201^Tl, ^99m^Tc and ^111^In separately.

## Results

### Phantom experiments

**Figure 3:**
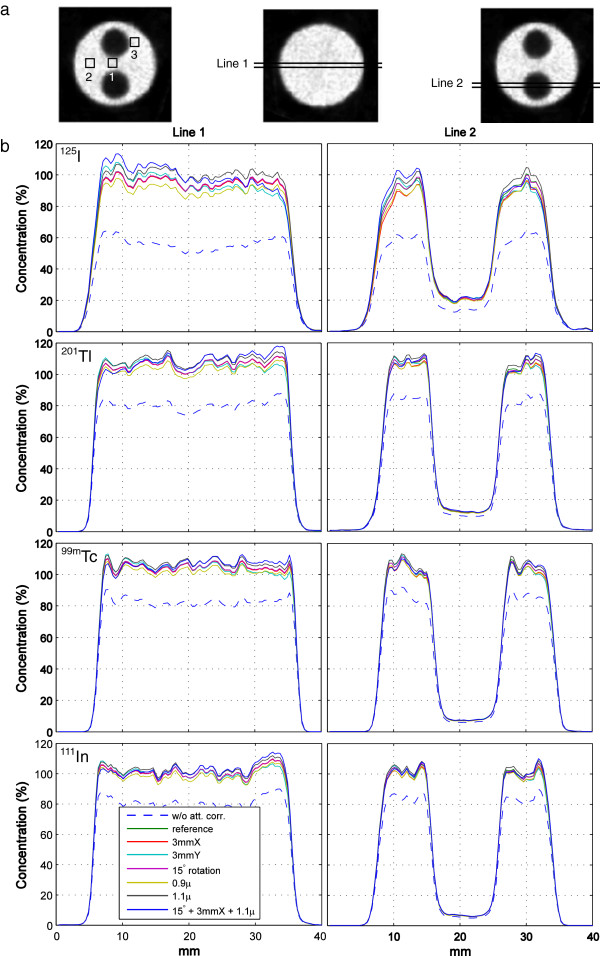
**VOIs and line profiles.** (**a**) VOI and line-profile definitions in phantom images. (**b**) Line profiles in phantom images with different attenuation map inaccuracies for four isotopes.

Three 3 mm × 3 mm × 11 mm volumes of interest (VOIs) were defined in heterogeneous slices of each phantom image, as shown in Figure 3a. Measured activities within the VOIs in the reference images and in the images corrected with inaccurate attenuation maps were calculated and compared. Changes in the measured VOI activities induced by attenuation map inaccuracies are listed in Table 1. With 1-mm shifts of the attenuation maps, the VOI activity changed less than 1.5% for ^125^I and 0.6% for ^201^Tl, ^99m^Tc and ^111^In. When the shifts increased to 3 mm, the changes increased to less than 4.5% for ^125^I and 1.7% for ^201^Tl, ^99m^Tc and ^111^In. With 15° rotation of the maps, the changes were less than 1.4% for ^125^I and 0.9% for ^201^Tl, ^99m^Tc and ^111^In. When the attenuation coefficients were altered by 10%, the activity in the VOIs changed less than 5.2% for ^125^I and 2.7% for ^201^Tl, ^99m^Tc and ^111^In. With the combination of rotation and shifts of the maps and altered attenuation coefficients, the VOI activity changed less than 6.2% for ^125^I and 3.0% for ^201^Tl, ^99m^Tc and ^111^In. The quantitative errors of the reference images are listed in the first data column of Table 1 for reasons of comparison.

**Table 1:** Quantitative errors and relative changes of VOI activities

**Isotope**	**VOI**	**Quantitative error**	**Changes induced by map inaccuracies (rotation, shift and/or*μ*change) with respect to references**							
			**0°**	**0°**	**0°**	**0°**	**15°**	**0°**	**0°**	**15°**
			**1 mm*X***	**3 mm*X***	**1 mm*Y***	**3 mm*Y***	**0 mm**	**0 mm**	**0 mm**	**3 mm*X***
			**1.0** ***μ***	**1.0** ***μ***	**1.0** ***μ***	**1.0** ***μ***	**1.0** ***μ***	**0.9** ***μ***	**1.1** ***μ***	**1.1** ***μ***
^125^I	1	−20.4	0.1	0.4	−0.5	−4.0	0.0	−4.9	5.2	5.3
	2	−11.8	0.7	1.1	−0.2	−1.0	−0.2	−4.8	5.0	6.2
	3	−7.9	−1.1	−4.0	−1.5	−4.5	−1.4	−4.2	4.3	−0.8
^201^Tl	1	−5.8	0.0	0.1	−0.1	−1.5	0.0	−2.6	2.7	2.7
	2	6.1	−0.4	−1.4	0.0	−0.3	−0.1	−2.5	2.6	3.0
	3	4.4	0.2	−0.3	0.6	1.4	−0.7	−2.3	2.3	0.0
^99m^Tc	1	−2.0	0.1	0.2	−0.2	−1.7	0.0	−2.2	2.2	2.1
	2	6.1	−0.3	−1.1	−0.1	−0.3	−0.1	−2.1	2.2	2.4
	3	−8.3	0.4	−0.2	0.5	1.3	−0.2	−1.8	1.9	0.2
^111^In	1	−4.7	0.0	0.1	−0.2	−1.4	0.0	−2.1	2.1	2.2
	2	−3.5	−0.4	−1.2	0.0	−0.3	−0.1	−2.0	2.0	2.5
	3	3.2	0.0	−1.1	0.4	0.9	−0.9	−1.9	1.9	0.6

Quantitative errors (in %) of VOI activities in reference phantom SPECT images were calculated with respect to dose calibrator measurements (1st data column), and relative changes (in %) in VOI activities due to attenuation map inaccuracies were calculated with respect to reference images (2nd to 9th data column).

In addition, the normalized root mean square deviation (NRMSD) was calculated voxel-wise between the reference images and the images corrected with inaccurate attenuation maps:

(3) $$\mathrm{NRMSD}=\sqrt{\frac{1}{n}\sum_{i=1}^{n}{\left(\frac{v_{i}-vr_{i}}{vr_{i}}\right)}^{2}}\times 100\%$$

where *v*_*i*_ is *i*th voxel inside the phantom chamber in an image corrected with an inaccurate map, and *vr*_*i*_ is the corresponding voxel in a reference image. *n* is the number of voxels inside the phantom chamber. The results are listed in Table 2. The largest NRMSDs caused by combined rotation, shifts and attenuation coefficient errors were 6.3%, 3.0%, 2.5% and 2.3% for ^125^I, ^201^Tl, ^99m^Tc and ^111^In, respectively.

**Table 2:** NRMSDs (in %) between phantom images corrected with inaccurate maps and with accurate maps

**Isotope**	**Rotation, shift and ×** ***μ***							
	**0°**	**0°**	**0°**	**0°**	**15°**	**0°**	**0°**	**15°**
	**1 mm*X***	**3 mm*X***	**1 mm*Y***	**3 mm*Y***	**0 mm**	**0 mm**	**0 mm**	**3 mm*X***
	**1.0** ***μ***	**1.0** ***μ***	**1.0** ***μ***	**1.0** ***μ***	**1.0** ***μ***	**0.9** ***μ***	**1.1** ***μ***	**1.1** ***μ***
^125^I	1.7	4.9	1.7	4.7	0.8	4.4	4.6	6.3
^201^Tl	0.8	2.2	0.7	2.2	0.4	2.4	2.5	3.0
^99m^Tc	0.6	1.8	0.6	1.8	0.4	2.1	2.1	2.5
^111^In	0.6	1.7	0.6	1.6	0.3	1.9	2.0	2.3

Line profiles through homogenous and heterogeneous slices (defined in Figure 3a) were created from images corrected with different attenuation maps and are plotted in Figure 3b. When the attenuation map was shifted, it is clear that the main changes in quantitation were located at the edges of the phantom along the shifting direction. The changes were small for ^201^Tl, ^99m^Tc and ^111^In imaging (≤5%), while for ^125^I it was larger (≤10%). Rotation of attenuation maps had almost no effect in homogenous slices for all four isotopes: the line profiles perfectly overlapped the reference profiles. In heterogeneous slices, the profiles slightly deviated (1% to 2%) from the reference profiles only close to the edges of the air chambers. When altering the attenuation coefficients by ±10%, the induced changes were on average about 2% to 3% for ^201^Tl, ^99m^Tc and ^111^In, and about 5% for ^125^I. Unlike shifting or rotating the maps, the changes in quantitation when the attenuation coefficients were altered resulted in global (but not uniform) over- or underestimations.

### Animal experiments

**Figure 4:**
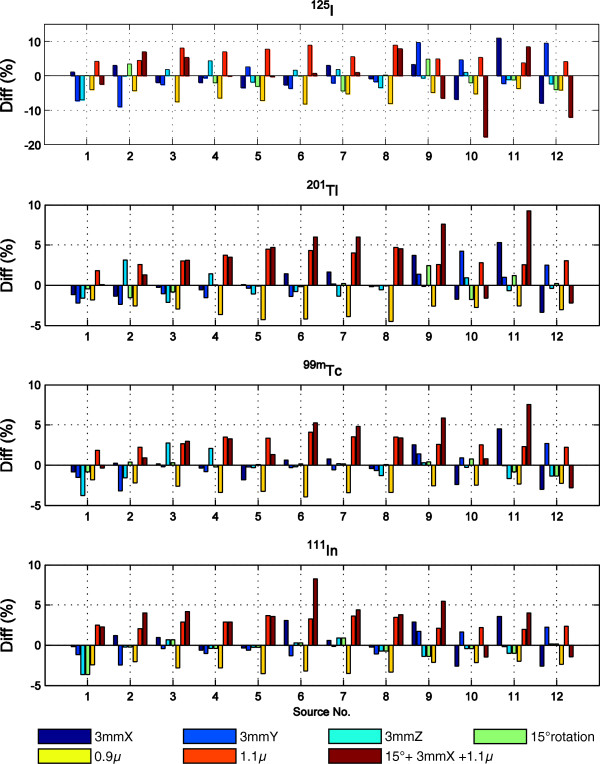
**Differences between source activities in rat SPECT images corrected with inaccurate attenuation maps and with accurate maps.** The differences are in percent (%).

In the rat SPECT images, non-overlapped spherical VOIs (diameter of about 15 mm) were created for each small source. The sources were completely enclosed in the centres of their corresponding VOIs. Activities in the VOIs were measured in the images corrected with different attenuation maps. Comparisons were made between measurements in the reference images and in the images corrected with inaccurate maps. Changes in the measured VOI activities induced by map inaccuracies are plotted in Figure 4, and the average absolute changes in percentage are listed in Table 3. With 1-mm shifts of the maps, the measured VOI activities changed 1.4 ± 1.1%, 0.6 ± 0.5%, 0.5 ± 0.4% and 0.5 ± 0.4% on average for ^125^I, ^201^Tl, ^99m^Tc and ^111^In, respectively, while these average changes increased to 3.6 ± 2.9%, 1.5 ± 1.2%, 1.3 ± 1.2% and 1.2 ± 1.0% with 3-mm shifts. When the maps were rotated by 15°, the changes were 2.1 ± 1.8%, 0.8 ± 0.8%, 0.5 ± 0.4% and 0.5 ± 0.8% on average. When altering the attenuation coefficients by 10%, the VOI activities changed 5.9 ± 1.8%, 3.3 ± 0.9%, 2.8 ± 0.7% and 2.7 ± 0.6% on average for the four isotopes, respectively. With combined rotated and shifted maps and altered attenuation coefficients, the average changes were 5.8 ± 5.4%, 4.4 ± 3.2%, 3.3 ± 2.2% and 3.9 ± 2.2% for ^125^I, ^201^Tl, ^99m^Tc and ^111^In, respectively. The quantitative errors of the reference images, as described in [16], are listed in the first data column of Table 3 for reasons of comparison.

**Table 3:** Average absolute quantitative errors and average absolute changes of source activities

**Isotope**	**Quantitative error**^**a**^	**Changes induced by map inaccuracies (rotation, shift and/or*μ*change) with respect to references**									
		**0°**	**0°**	**0°**	**0°**	**0°**	**0°**	**15°**	**0°**	**0°**	**15°**
		**1 mm*X***	**3 mm*X***	**1 mm*Y***	**3 mm*Y***	**1 mm*Z***	**3 mm*Z***	**0 mm**	**0 mm**	**0 mm**	**3 mm*X***
		**1.0** ***μ***	**1.0** ***μ***	**1.0** ***μ***	**1.0** ***μ***	**1.0** ***μ***	**1.0** ***μ***	**1.0** ***μ***	**0.9** ***μ***	**1.1** ***μ***	**1.1** ***μ***
^125^I	2.1 ± 6.5	1.5 ± 1.1	3.9 ± 3.1	1.6 ± 1.2	4.6 ± 3.3	0.9 ± 0.9	2.3 ± 1.9	2.1 ± 1.8	5.8 ± 1.7	6.1 ± 1.9	5.8 ± 5.4
^201^Tl	3.3 ± 2.5	0.6 ± 0.6	1.7 ± 1.6	0.6 ± 0.4	1.5 ± 1.2	0.5 ± 0.5	1.2 ± 0.8	0.8 ± 0.8	3.2 ± 0.8	3.3 ± 0.9	4.4 ± 3.2
^99m^Tc	2.0 ± 2.5	0.6 ± 0.5	1.5 ± 1.4	0.4 ± 0.4	1.0 ± 1.0	0.5 ± 0.4	1.3 ± 1.2	0.5 ± 0.4	2.8 ± 0.6	2.9 ± 0.7	3.3 ± 2.2
^111^In	2.0 ± 2.7	0.6 ± 0.4	1.6 ± 1.3	0.4 ± 0.3	1.2 ± 0.8	0.3 ± 0.4	0.8 ± 1.0	0.5 ± 0.8	2.7 ± 0.6	2.8 ± 0.6	3.9 ± 2.2

Average absolute quantitative errors (in %) of source activities in reference rat SPECT images were calculated with respect to dose calibrator measurements (1st data column), and average absolute changes (in %) in source activities due to attenuation map inaccuracies were calculated with respect to reference images (2nd to 11th data column). ^a^Obtained from [16].

## Discussion

We previously have shown that quantitative differences between uniform and non-uniform attenuation correction are quite small in micro-SPECT [16]. In this study, the influence of other attenuation map imperfections (i.e. misregistration and global errors in attenuation coefficient) on the quantitative accuracy of attenuation-corrected SPECT was investigated. For this purpose, we mimicked misregistration between micro-SPECT and micro-CT, and global deviation of attenuation coefficients. A mismatch of a few millimetres is already a big error for image registration in small-animal imaging: e.g. Ji et al*.*[31] showed that when using a proper calibration method for co-registration and a special bed mounting interface, the accuracy of small-animal SPECT-CT registration can reach sub-millimetre accuracy without using extra markers. Deviation of attenuation coefficients can be caused by the errors in CT voxel values (in HU) or from the method that translates HU numbers into linear attenuation coefficients. The system error of CT (in HU) can be eliminated by re-calibration with a water phantom, and other effects such as ring artefacts and beam-hardening effects can be well controlled and suppressed with proper reconstruction algorithms. Many reliable models for translating HU to attenuation coefficients for attenuation correction in SPECT and positron emission tomography have been proposed and evaluated, e.g. in [32,33]. Therefore, the level of the inaccuracies set in our present investigation is expected to exceed those applied in most real small-animal studies.

For both phantom and animal experiments, the changes in image quantitation due to strong attenuation map inaccuracies were less than about 5%, except for ^125^I (worst case approximately 10%), which can be attributed to ^125^I attenuation that is more prominent.

**Figure 5:**
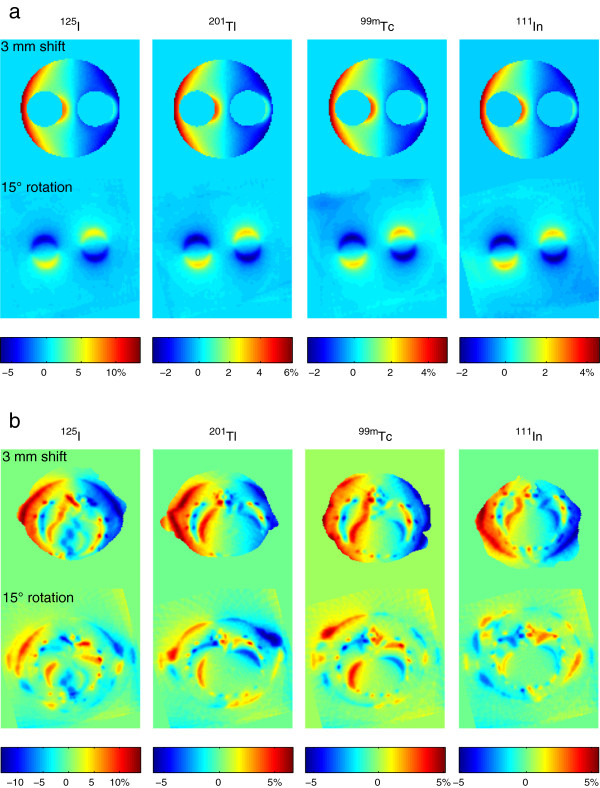
**Relative differences in attenuation due to 3-mm shifts or 15° rotation.** (**a**) Phantom heterogeneous slices and (**b**) rat thoracic slices.

In the phantom studies, quantitation changes due to attenuation map shifts were small inside the phantom, but increased close to the outer edge to a maximum of about 5% for ^125^I and about 2% to 3% for ^201^Tl, ^99m^Tc and ^111^In. Although sharp attenuation and activity changes exist at the edges of the air chambers in the phantom, the changes in TF maps are rather smooth. As a result, quantification errors due to misregistration stayed also small in these regions. Quantitation changes caused by map rotation were smaller than the changes by shifts, and because the axis of rotation was closely aligned with the axis of the phantom, the changes only existed close to the air chambers. This may be relevant for quantification in e.g. tumours that are close to lungs or in cardiac imaging. These changes of measured activity concentration were consistent with the relative differences between the reference TF maps and the shifted or rotated maps, as shown in Figure 5a. When imaging the small sources in the rat cadavers, the activity changes in a subset of sources due to shifts and rotation were a bit larger than changes in the phantom, which can be explained by the effects of non-uniform attenuation close to the sources. Again, the influence of rotation on map accuracy was smaller than that of shifts, as shown in the rat thoracic slices in Figure 5b. Since both large shifts and rotation have limited effects on quantitative accuracy in our emulation, we believe that the typical misalignments between micro-SPECT and micro-CT caused by sub-optimal registration and/or animal movement during scans will not often have a prominent impact to SPECT quantitative accuracy.

**Figure 6:**
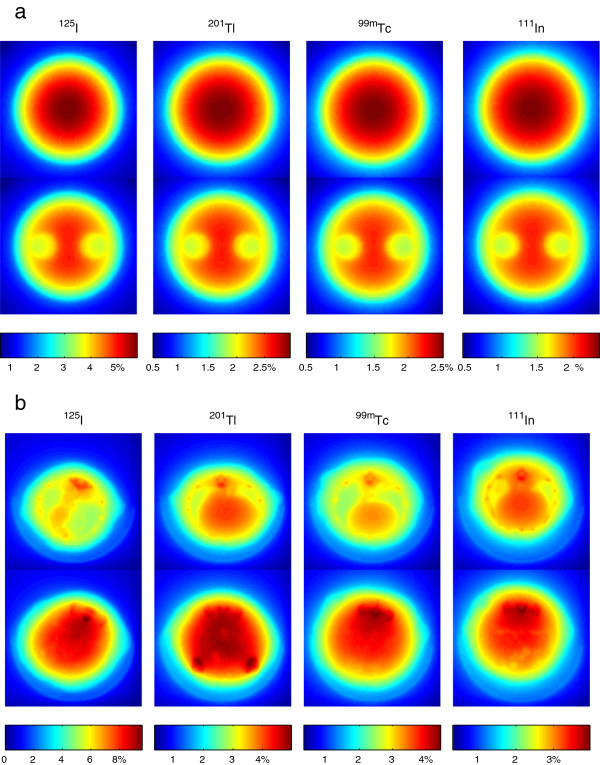
**Relative differences in attenuation due to multiplying attenuation coefficients by 1.1.** (**a**) Two different phantom slices and (**b**) two different slices through each rat.

With attenuation coefficients globally altered by ±10%, similar global over- or underestimation was observed in both phantom and animal studies. We also examined the relative differences between the reference TF maps and the ones derived from 10% increased attenuation coefficients. Figure 6a shows the differences in two transaxial slices of phantom TF maps for each isotope. One slice was from the part of the phantom without air chambers, and the other one was from the part that contains the two air chambers. Figure 6b shows the differences in two slices through the rat TF maps: one slice across the thorax and the other across the abdomen of each rat. It is clear that with a global change of the attenuation coefficients, the changes in the TF maps are not homogenous. The effects are larger around bone areas as shown in Figure 6b and are smaller at areas with low density such as the air chambers in Figure 6a and lungs in Figure 6b. Ostensibly, this implies that the attenuation coefficient accuracy in and around the bones may play a relatively important role in the accuracy of SPECT attenuation correction. However, the contribution of bone attenuation is limited in the integral in Equation 1 for small animals so that effects on quantification would not spread widely, as shown in the ‘Results’ section and in Figure 6b.

Without attenuation correction, activities were roughly underestimated by 40% for ^125^I and 20% for ^201^Tl, ^99m^Tc and ^111^In. Even the largest inaccuracies in attenuation maps investigated here effectively took away only one eighth of the benefit from the attenuation correction. Since accuracy of the attenuation coefficients and alignment between micro-SPECT and micro-CT in practice are usually better than in the worst situations emulated in the present study, effects of attenuation map inaccuracies on micro-SPECT quantification will usually be small.

## Conclusions

For the more commonly used SPECT isotopes like ^201^Tl, ^99m^Tc and ^111^In, misalignments up to a few millimetres or in the order of 10° to 15° between micro-SPECT and micro-CT, and/or global attenuation coefficient inaccuracies in the range of ±10% have quite small effects on micro-SPECT quantification. Therefore, we conclude that micro-SPECT quantification is quite robust to imperfections in attenuation maps for most applications.

## Abbreviations

CT: Computed tomography; NRMSD: Normalized root mean square deviation; SPECT: Single-photon emission computed tomography; TF: Transmitted fraction; VOI: Volume of interest.

## Competing interests

Freek J. Beekman is a stockholder of and gets honoraria and grant/research support from MILabs. Hugo A. Gratama van Andel is an employee of MILabs. The other authors declare that they have no competing interests.

## Authors’ contributions

CW designed the study, performed the experiments, analysed and interpreted the data, and drafted and revised the manuscript. HAGvA analysed and interpreted the data and revised the manuscript. PL co-performed the experiments and revised the manuscript. OCB participated in the study design and coordination and revised the manuscript. FJB conceived of and designed the study, participated in its coordination and drafted and revised the manuscript. All authors read and approved the final manuscript.
